# Ameliorative effect of morin on diclofenac-induced testicular toxicity in rats: An investigation into different signal pathways

**DOI:** 10.22038/ijbms.2025.79735.17270

**Published:** 2025

**Authors:** Hasan Şimşek, Nurhan Akaras, Cihan Gür, Sefa Küçükler, Mustafa İleritürk, Fatih Mehmet Kandemir

**Affiliations:** 1 Department of Physiology, Faculty of Medicine, Aksaray University, Aksaray, Türki̇ye; 2 Department of Histology and Embryology, Faculty of Medicine, Aksaray University, Aksaray, Türki̇ye; 3 Department of Medical Laboratory Techniques, Vocational School of Health Services, Atatürk University, Erzurum, Türki̇ye; 4 Department of Veterinary Biochemistry, Faculty of Veterinary, Atatürk University, Erzurum, Türki̇ye; 5 Department of Animal Science, Horasan Vocational College, Atatürk University, Erzurum, Türki̇ye; 6 Department of Medical Biochemistry, Faculty of Medicine, Aksaray University, Aksaray, Türki̇ye

**Keywords:** Apoptosis, Diclofenac, Endoplasmic reticulum – stress, Inflammation, Morin, Oxidative stress

## Abstract

**Objective(s):**

Diclofenac (Diclo) is a therapeutic agent used in the treatment of pain and inflammatory diseases, but it is also toxic to the human body. Morin is a flavonoid found naturally in plants and has many biological and pharmacological activities, including anti-inflammatory, anti-oxidant, and anticancer activities. This study aimed to investigate the efficacy of Morin in Diclo-induced testicular toxicity.

**Materials and Methods:**

Morin (50 mg/kg and 100 mg/kg) was administered orally for five days, while Diclo was administered intraperitoneally at 50 mg/kg on days 4 and 5. Biochemical, molecular, and histological methods were used to investigate oxidative stress, inflammation, apoptosis, and endoplasmic reticulum (ER) stress damage indicators in testicular tissue.

**Results:**

Morin treatment attenuated Diclo-induced oxidative stress damage by increasing anti-oxidant levels (SOD, CAT, GPx, GSH, Nrf-2, HO-1, and NQO1) and decreasing MDA levels, an indicator of lipid peroxidation. Morin reduced levels of the inflammatory mediators NF-κB protein. Increases in apoptotic Bax and Caspase-3 by Diclo were reduced by Morin, while decreased antiapoptotic Bcl-2 level was increased. Morin reduced Diclo-induced ER stress injury by decreasing ATF-6, PERK, IRE1, GRP-78, and CHOP levels. Also, Diclo decreased COX-2 levels.

**Conclusion:**

Overall, Morin may be an effective treatment of choice for testicular tissue damage associated with Diclo toxicity and may reduce the level of damage.

## Introduction

The most commonly used drug derivatives in treating pain and inflammation in the clinic are non-steroidal anti-inflammatory drugs (NSAIDs), and diclofenac (Diclo) has an essential place among them (1). Millions of people use Diclo to treat pain, inflammation, degenerative joint disease, rheumatoid arthritis, dysmenorrhea, and trauma. The amount of Diclo consumed globally in a year is approximately 940 tons (2). Diclo is a weak acid that specifically inhibits the cyclooxygenase-2 (COX-2) enzyme. The peak concentration of Diclo in plasma occurs between 10–120 min, depending on the dose and form of administration and individual patient parameters such as gastrointestinal pH (3). Testicular tissue is one of the target tissues in Diclo toxicity due to its high oxygen consumption, high polyunsaturated fatty acids, and low anti-oxidant capacity compared to other tissues in the body (4). It is now a known fact that NSAIDs also adversely affect reproductive functions. Like other NSAIDs, Diclo can have a toxic effect by causing degenerative changes in the testis, epididymis, and accessory sex glands. Diclo causes oxidative stress and fragmentation of genomic DNA because it accelerates the synthesis of reactive oxygen species (ROS) (5). Abnormal spermatozoa resulting from high levels of ROS production is one of the etiologies of infertility, a defect in the male reproductive system. Lipid peroxidation in the membrane of sperm cells is the basis of ROS-induced damage (6). Therefore, studies have reported that natural anti-oxidant compounds - especially herbal active ingredients - may be effective in testicular toxicity caused by toxic agents (7).

Nutrients with anti-oxidant properties have been actively investigated in studies to reduce the toxic effects of drugs (8). Among these anti-oxidants, flavonoids are high-anti-oxidant compounds in plant and bee products (9, 10). Morin (3,5,7,2′,4′-pentahydroxyflavone) is a natural bioflavonoid with anti-oxidant, anti-inflammatory, anti-diabetic, anti-carcinogenic, neuroprotective, and antiproliferative effects (11). Morin shows its anti-oxidant properties by counteracting ROS-induced damage (12). In addition to all these properties, the most significant feature of Morin that distinguishes it from other anti-oxidants is that it has limited toxic effects at high doses in studies on experimental animals (13).

The present study was designed to examine the efficacy of Morin against Diclo-induced rat testicular toxicity. To determine this efficacy, oxidative stress, inflammation, endoplasmic reticulum (ER) stress, and apoptotic damage parameters were examined.

## Materials and Methods

### Chemicals

Diclo (Abdi Ibrahim, 75 mg/3 ml) and Morin (Sigma, 302.24 g/mol; CAS no: 654055-01-3) were purchased commercially. The other chemicals used in this study were obtained from Sigma–Aldrich Co. (St. Louis, MO, USA). Morin was dissolved in physiological saline.

### Animals and groups

In the experiment, 35 male Wistar albino rats weighing 220–250 g and aged 10–12 weeks were used. The animals were kept in cages in a controlled room with a constant temperature of 24-25 °C and a twelve-hour dark-light cycle (07:00-19:00 light; 19:00-07:00 dark). They were given unlimited access to water and standard chow. Experiments were started after the rats were allowed to rest in their cages for a week and adapted to the environment. Rats were obtained from the KONÜDAM Experimental Medicine Application and Research Center (Konya/Türkiye). Rats were randomly divided into five groups (n=7);

1: Control Group: Saline was administered orally daily for 5 days. 

2: Morin Group: 100 mg/kg orally once daily for five days.

3: Diclofenac Group (Diclo): Diclo 50 mg/kg was administered intraperitoneally on days 4 and 5.

4: Diclofenac + Morin 50 mg/kg (Diclo+Morin 50): Diclo 50 mg/kg was administered intraperitoneally on days 4 and 5. Morin was administered orally at 50 mg/kg for 5 days.

5: Diclofenac + Morin 100 mg/kg (Diclo+Morin 100): Diclo 50 mg/kg was administered intraperitoneally on days 4 and 5. Morin 100 mg/kg was administered orally once daily for 5 days.

Studies in the literature have been used to determine the doses of Diclo and Morin (12, 14).

### Sample collection

Twenty-four hours after the last administration (day 6), the animals were decapitated under mild sevoflurane anesthesia. Testicular tissues were removed and cleaned from surrounding tissues. A testicle was placed in liquid nitrogen for biochemical, molecular, and western blot analysis. The other specimen was placed in 10% formaldehyde for histopathological analysis.

### Examination of oxidative stress in testicular tissues

A homogenizer (Tissue Lyser II, Qiagen, The Netherlands) was used to prepare homogenates from testicular tissues by placing the tissues in a 1.15% KCl solution. Glutathione peroxidase (GPx) activity was determined using the Lawrence & Burk method (15), superoxide dismutase (SOD) activity was determined using the Sun *et al*. method (16), catalase (CAT) activity was determined using the Aebi method (17), glutathione (GSH) level was determined using the Sedlak & Lindsay method (18). MDA level was determined using the Placer *et al*. method (19). The protein content of testicular tissues was determined using the Lowry *et al*. method (20).

### Gene expression analysis

QIAzol Lysis Reagent (79306; Qiagen) was used for total RNA isolation from testicular tissues in all groups. All procedures for RNA isolation were performed according to the manufacturer’s instructions. An iScript cDNA Synthesis Kit (Bio-Rad) was used to obtain cDNA from RNA, and the manufacturer’s instructions were followed step by step. The cDNAs obtained were used to determine the relative mRNA transcript levels of COX-2, Bcl-2-associated X (Bax), B-cell lymphoma 2 (Bcl-2), cysteine-aspartic acid protease-3 (Caspase-3), Activating transcription factor 6 (ATF-6), Protein Kinase RNA-Like ER Kinase (PERK), Inositol-requiring enzyme 1 (IRE1), Glucose-regulated protein (GRP-78), C/EBP homologous protein (CHOP), Nuclear factor erythroid 2-related factor 2 (Nrf-2), Heme oxygenase-1 (HO-1), and NAD(P)H Quinone Dehydrogenase 1 (NQO1) primer sequences are presented in Table 1. In these procedures, cDNAs reacted with iTaq Universal SYBR Green Supermix (BIO-RAD) and primers for the respective genes in Rotor-Gene Q (Qiagen). The manufacturer’s instructions were followed when preparing the reaction cycles. After the cycles were completed, the CT values obtained from the device were normalized to ß-Actin using the 2^ −ΔΔC T^ method (21).

### Western blot analysis

Western blot analyses were performed on testicular tissues, similar to the method previously performed in our laboratory (22). In summary, some samples taken from the testicular tissues, which were pulverized by crushing them in a mortar with liquid nitrogen, were homogenized in RIPA buffer and then centrifuged (16000 g, 20 min). The total protein level was measured from the obtained supernatants using the protein BCA assay kit (Thermo Fischer). Supernatants treated with Laemmli sample buffer were separated on a 10% sodium dodecyl sulfate-polyacrylamide gel electrophoresis containing 30 μg of protein in each well. After ​​​​​​​the proteins with determined molecular sizes were transferred to PVDF membranes, the membrane was blocked with 4% Bovine serum albumin (BSA) (dissolved in phosphate-buffered saline containing 0.1% tween (PBS-T)) for 90 min. After this process, primary antibodies were added and incubated overnight. It was then incubated with goat anti-mouse IgG secondary antibody conjugated to HRP for 90 min (1:2,000 dilution). At the end of the period, the membranes were washed with PBS-T (5 times, 5 min each time). Densitometric analysis was performed in the ImageLab program (Bio-Rad, Hercules, USA) using BioRad Clarity Max ECL substrate (Bio-Rad, Hercules, USA) to visualize the bands. At least three repeated measurements were taken for each sample. 

### Histopathological evaluations

Testicular tissues were kept in 10% neutral buffered formalin for approximately 72 hr for fixative purposes. Testicular tissues were kept overnight under running water and dehydrated by passing through an ascending series of alcohols. They were then made transparent in xylene and kept in molten paraffin. Paraffin-embedded tissues were sectioned at a thickness of 5 μm using a microtome and stained with hematoxylin and eosin (H&E). The stained testicular tissues were examined using a binocular Olympus Cx43 light microscope (Olympus Inc., Tokyo, Japan) and photographed with an EP50 camera (Olympus Inc., Tokyo, Japan). According to histopathologic findings (tubular wall thickness, edema in intertubular spaces, degeneration and necrosis in spermatogonia, number of spermatozoa in the lumen), sections were classified as absent (-), mild (+), moderate (+ + ) and severe (+ + +).

### Statistical analysis

Data were analyzed using IBM SPSS. Statistical values were determined based on mean ± standard deviation. One-way ANOVA/Tukey’s *post hoc* tests were used for multiple comparisons. In real-time PCR analyses, each sample was run in triplicate, and results are presented as mean ± SD. *P*<0.05 was considered statistically significant.

### Effect of Diclo and Morin on oxidative stress parameters

**Figure 1:**
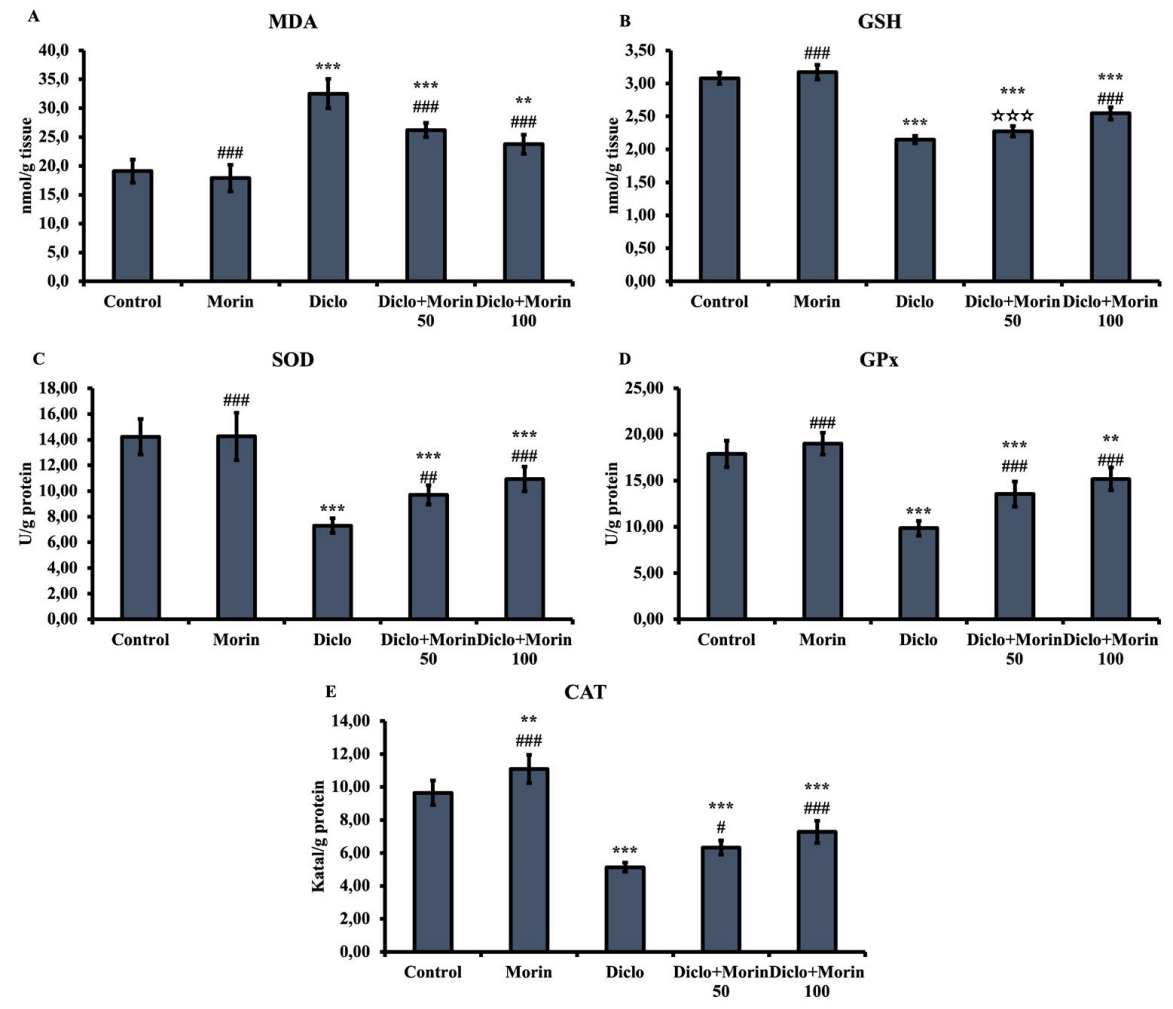
Effects of diclofenac and Morin administrations on MDA and GSH levels and SOD, GPx, and CAT activity in testicular tissue of rats

To determine oxidative stress damage, lipid peroxidation indicator MDA was measured as an oxidant, and SOD, CAT, GPX enzyme activity, and GSH level were measured as anti-oxidants (Figure 1). Compared to the control group, MDA increased (*P*<0.001), while all anti-oxidants decreased in the Diclo group (*P*<0.001). When Morin was applied together with Diclo, these changes were reversed. Compared to the Diclo group, all these changes are more prominent at 100 mg/kg in morin co-treatment (*P*<0.001).

### Effect of Diclo and Morin on Nrf-2, HO-1, and NQO1 mRNA transcription levels

**Figure 2:**
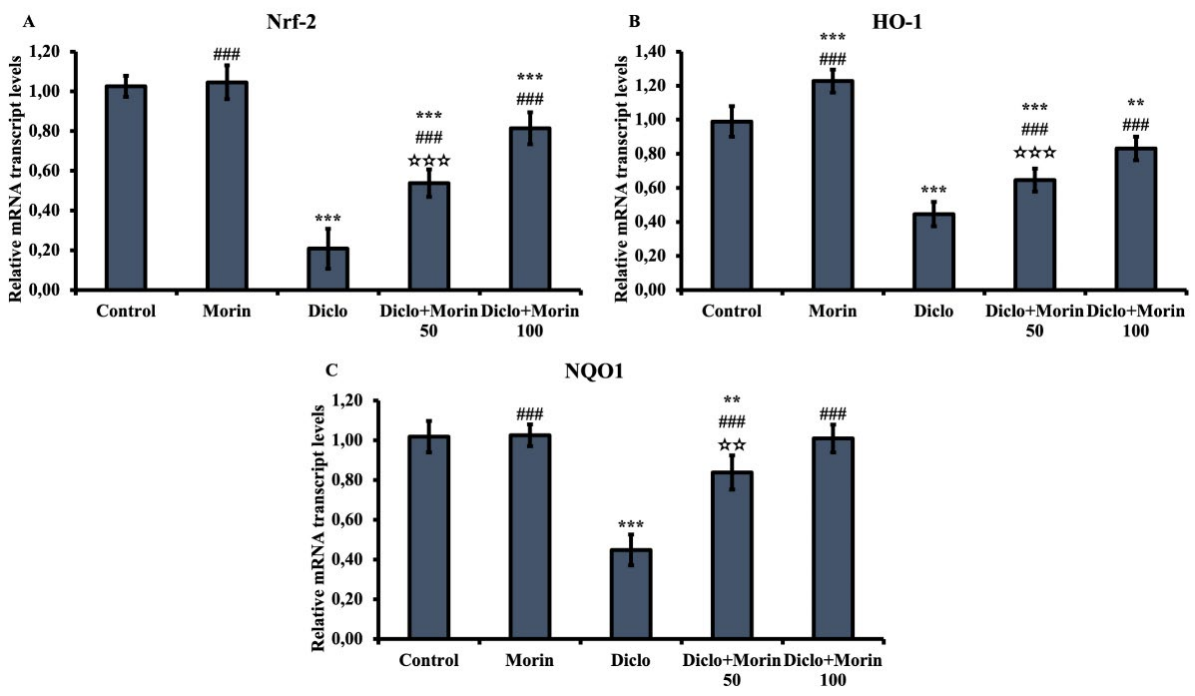
Effects of Diclo and Morin administrations on Nrf-2, HO-1, and NQO1 mRNA transcription levels in testicular tissue of rats

There was a decrease in Nrf-2, HO-1, and NQO1 mRNA transcription levels in the Diclo group compared to the control group (*P*<0.001). With morin co-treatment, this situation changed, and an increase occurred compared to Diclo (*P*<0.001). Morin at 100 mg/kg was more effective (*P*<0.001 for Nrf-2 and HO-1; *P*<0.01 for NQO1) (Figure 2).

### Effect of Diclo and Morin on ATF-6, PERK, IRE1, GRP-78, and CHOP mRNA transcription levels

**Figure 3:**
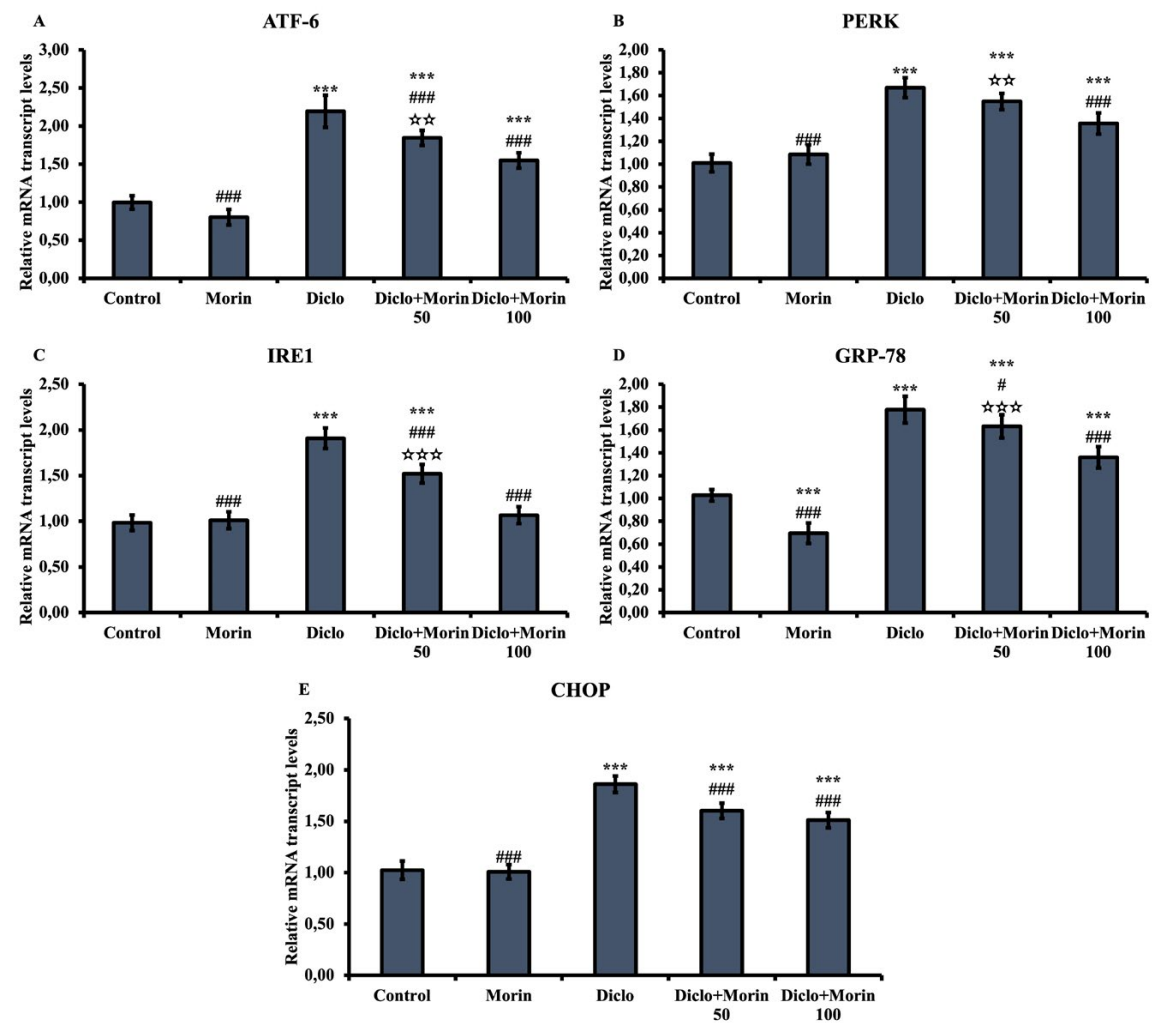
Effects of diclofenac and Morin administrations on ATF-6, PERK, IRE1, GRP-78, and CHOP mRNA transcription levels in testicular tissue of rats

To determine the level of ER damage, ATF-6, PERK, IRE1, GRP-78, and CHOP mRNA transcription levels were measured (Figure 3). All these parameters increased in the Diclo group compared to the control group (*P*<0.001). On the other hand, Morin treatment reversed this situation and reduced the level of these parameters. In particular, this reduction was much more effective in the 100 mg/kg dose of Morin than in the Diclo group (*P*<0.001).

### Effect of Diclo and Morin on Bax, Bcl-2, and caspase-3 mRNA transcription levels

**Figure 4:**
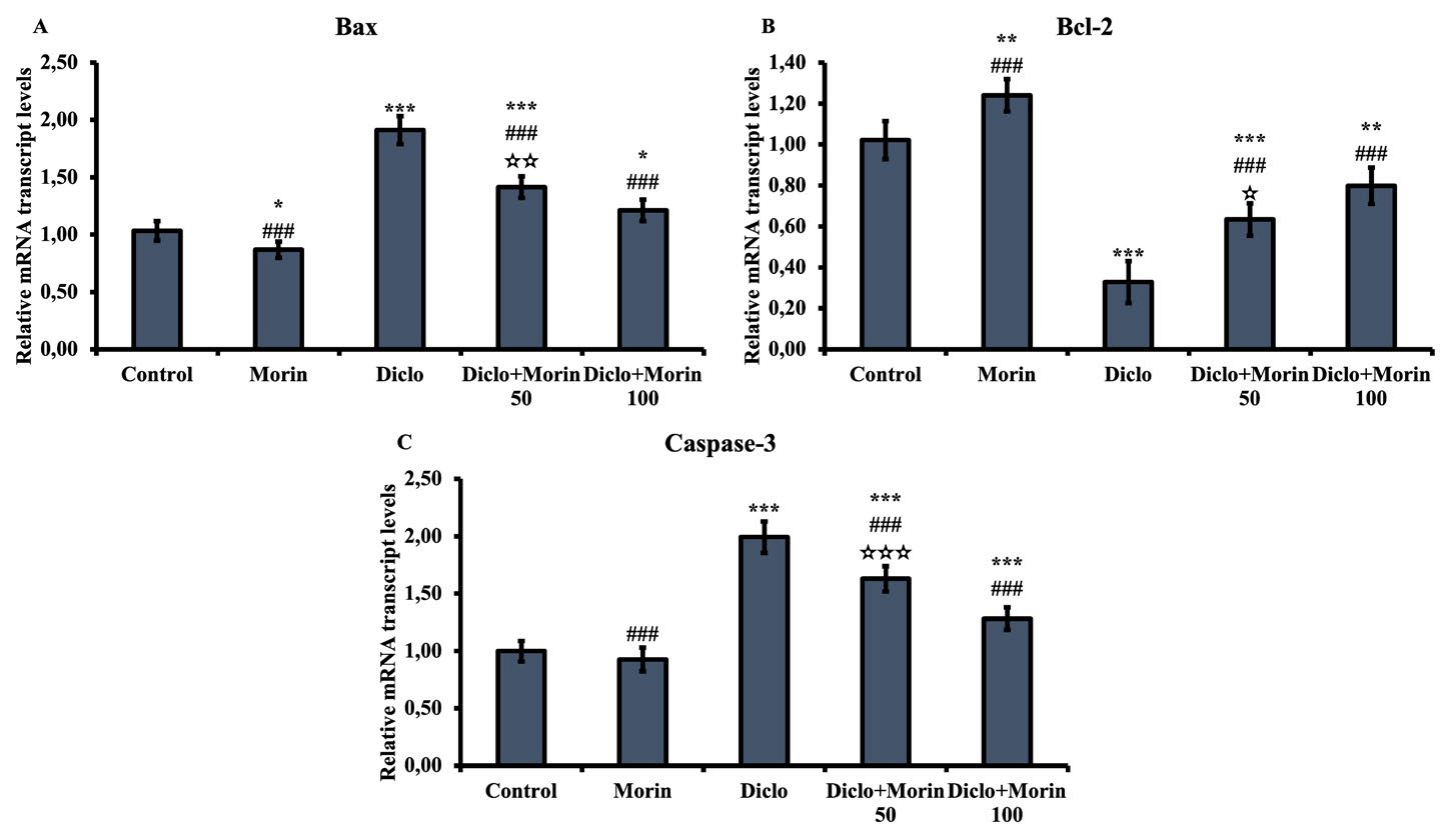
Effects of diclofenac and Morin administrations on Bax, Bcl-2, and caspase-3 mRNA transcription levels in testicular tissue of rats

To determine the level of apoptotic damage, Bax (apoptotic), Bcl-2 (antiapoptotic), and caspase-3 (apoptotic) mRNA transcription levels were measured (Figure 4). Bax and caspase-3 increased while Bcl-2 decreased in the Diclo group compared to the control group (*P*<0.001). With Morin application with Diclo, this situation moved in the opposite direction, and Bax and caspase-3 decreased while Bcl-2 increased (*P*<0.001 at both doses). Morin was more effective at 100 mg/kg with varying significance.

### Effect of Diclo and Morin on COX-2 mRNA transcription levels

**Figure 5:**
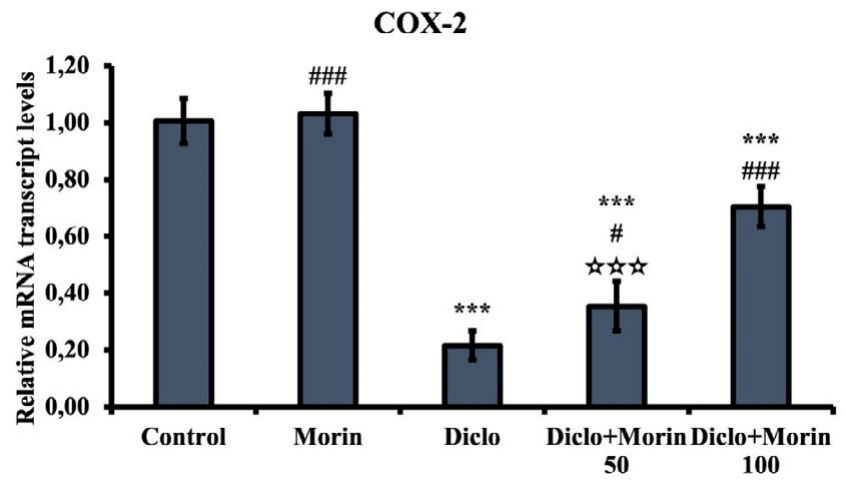
Effects of diclofenac and Morin administrations on COX-2 mRNA transcription levels in testicular tissue of rats

Since Diclo is a selective inhibitor of COX-2, testicular tissue COX-2 mRNA transcription level was also measured (Figure 5). Indeed, COX-2 was reduced in the Diclo group compared to the control group (*P*<0.001). COX-2 mRNA transcription levels increased with Morin administration (50 mg/kg: *P*<0.05; 100 mg/kg: *P*<0.001). Morin was more effective at 100 mg/kg than 50 mg/kg (*P*<0.001).

### Effect of Diclo and Morin on NF-κB, caspase-3, and Bax protein levels

**Figure 6:**
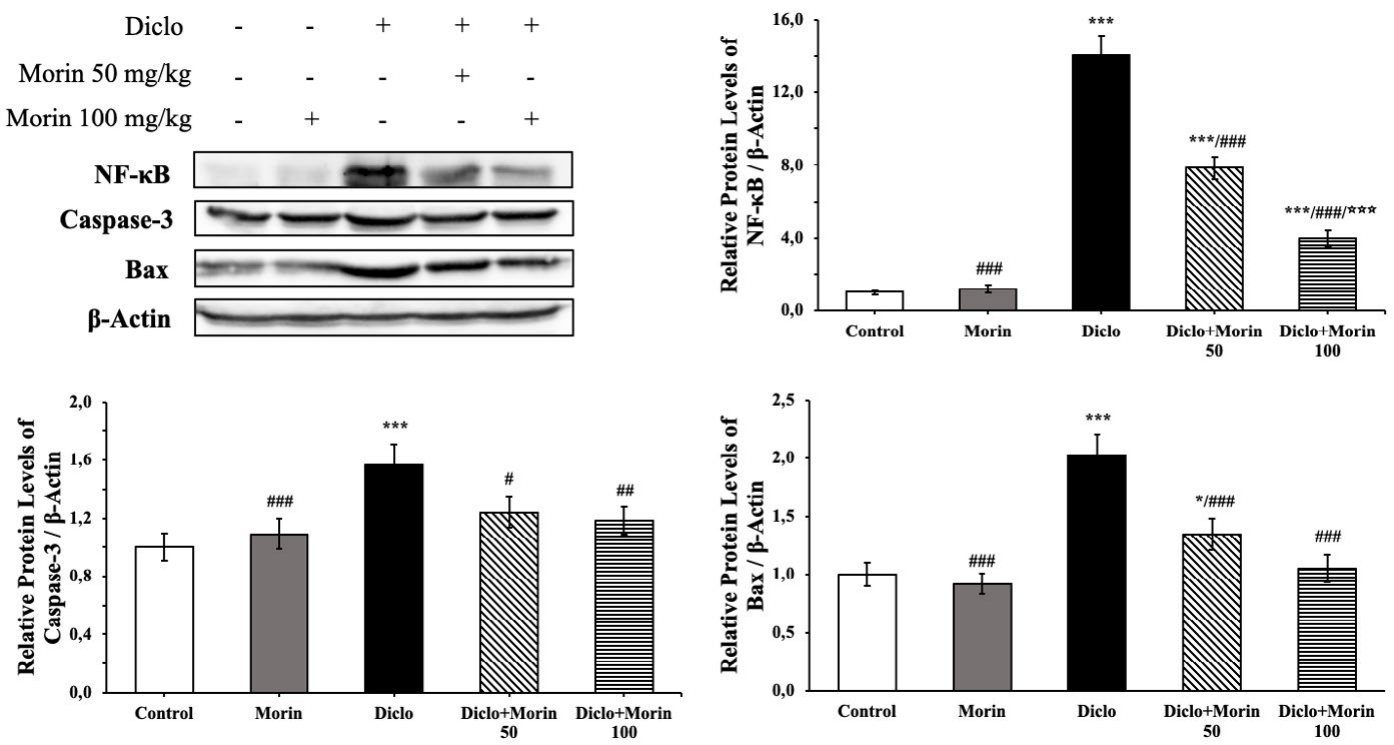
Effects of diclofenac and Morin administrations on NF-κB, caspase-3, and Bax protein levels in testicular tissue of rats

NF-κB, caspase-3, and Bax protein levels were also analyzed from testicular tissues (Figure 6). Inflammation marker (NF-κB) and apoptotic factors (caspase-3 and Bax) increased in the Diclo group compared to the control group (*P*<0.001). Compared to the Diclo group, Morin 50 mg/kg decreased NF-κB (*P*<0.001), Bax (*P*<0.001), and caspase-3 (*P*<0.05), whereas this decrease was more pronounced in the 100 mg/kg (*P*<0.001).

### Histopathology findings

**Figure 7:**
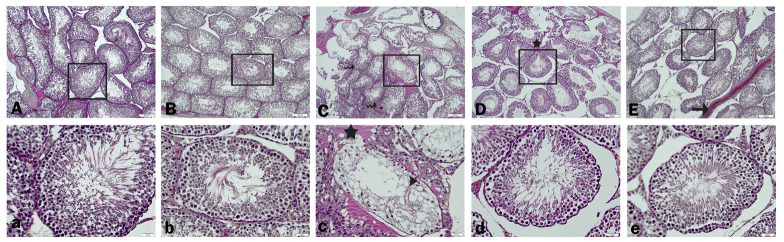
Representative light micrographs from H&E-stained testicular tissue sections in groups

Histopathologic findings are summarized in Figure 7 and Table 2. When the testicular tissue findings of the control and Morin group were evaluated, the morphology and wall thickness of the seminiferous tubules were regular, and the number of spermatozoa in the lumen was high (Figure 7Aa, 7Bb). However, the tubule walls of the sections belonging to the Diclo group were thinned, and tubular atrophy and severe edema in the intertubular area were observed. In addition, loss of tubule cells, necrotic and degenerative changes in spermatogonia, and a severe decrease in the number of spermatozoa in the lumens were observed (Figure 7Cc). In the Diclo+Morin 50 and Diclo+Morin 100 groups, mild degeneration of spermatogonia, mild edema in the intertubular area in the low dose treatment group, no edema in the high dose group and spermatozoa in the tubule lumens were increased compared to the Diclo group. In addition, the thickening of the seminiferous tubule walls was observed (Figure 7Dd, 7Ee).

## Discussion

Studies on Diclo, one of the NSAIDs, have reported harmful effects on human and experimental animal organs and tissues (23). Widely used and effective in the treatment of inflammation and pain, Diclo also has serious side effects (24). Oxidative stress has been reported to be one of the factors causing Diclo toxicity (25). Morin is a natural flavonoid found in plants of the *Moraceae* family and is well tolerated even with long-term use (26). Morin has a protective effect by reducing apoptosis and ROS production (27). In this study, the effects of Morin on Diclo-induced testicular toxicity were investigated.

In physiological conditions of healthy cells, there is a balance between oxidants such as ROS and anti-oxidants. However, oxidative stress may occur due to the imbalance between this balance factor shifting to the side of oxidants (28,29). The increase in free radicals leads to lipid peroxidation in the cell. Because lipid density is high in the cell membrane (7, 30). MDA is the most prominent indicator of lipid peroxidation caused by oxidative stress (31, 32). The body forms a defense system against oxidative stress damage by activating various anti-oxidant enzymes such as SOD, CAT, and GPx and non-enzymatic anti-oxidants such as GSH (33-35). In studies conducted with NSAIDs, it was found that ROS levels increased (36, 37). Testicular tissue is highly susceptible to oxidative stress as both spermatogenesis and steroidogenesis are affected by ROS (38, 39). In rats, Diclo caused a decrease in anti-oxidant defense systems SOD, CAT, and GPx activities and GSH levels in the testis and epididymis. Thus, Diclo inhibits anti-oxidant defense systems in the testis and epididymis and may trigger oxidant levels to reach dangerous levels and oxidative stress damage (2). In the present study, Diclo contributed to oxidative stress damage by decreasing anti-oxidant defense systems SOD, CAT, GPx activities, and GSH levels. On the other hand, Diclo increased the oxidant MDA level. Morin treatment decreased oxidative stress damage by increasing SOD, CAT GPX activities, and GSH levels and decreasing MDA levels. Morin at a dose of 100 mg/kg further reduced oxidative damage. Co-administration of Morin reduced Diclo-induced oxidative damage in testicular tissues in a dose-dependent manner.

Another indicator of oxidative stress damage is the Nrf2/HO-1 signaling pathway (40, 41). Nrf-2 is a transcription factor in defense against cell oxidative stress damage (42). With the increase in oxidative stress, Nrf-2, located in the cytoplasm, separates from the complex it forms with Keap-1 and translocates towards the nucleus (43). It then stimulates the transcription of enzymes such as HO-1 and NQO1 (44, 45). HO-1 is effective against ROS-induced oxidative stress damage in tissues, while NQO1 involves protection against both natural and exogenous quinones, maintenance of endogenous anti-oxidants, and stabilization of p53 protein (34). Varışlı *et al*. (1) reported that Diclo suppressed Nrf-2, HO-1, and NQO1 mRNA transcription in different tissues in their study. A study (46) reported that Morin increased Nrf-2 and HO-1 mRNA transcription levels in different tissues. In the present study, Diclo decreased Nrf-2, HO1, and NQO1 mRNA transcription levels in testicular tissue and decreased the level of anti-oxidant defense through this pathway. On the other hand, this situation was reversed with Morin administration, and Nrf-2, HO1, and NQO1 mRNA transcription levels increased. Thus, Morin reduced Diclo-induced oxidative stress damage in testicular tissue via the Nrf-2 pathway.

COX-2 is a factor involved in many physiological processes. In these processes, different mediators (growth factors, lipopolysaccharide, interleukin-1β, and tumor necrosis factor-alpha) affect COX-2 expression (47). It is now well known that Diclo has a selective inhibitory effect on COX-2 (48). The present study also measured COX-2 mRNA transcription level to determine Diclo effects in testicular tissue. Indeed, the COX-2 mRNA transcription level was significantly decreased in the Diclo-treated group. This may also prove that Diclo is effective in testicular tissue.

Apoptosis (programmed cell death) is a biological process that enables the organism to eliminate cells that are damaged in developmental stages, in the maintenance of homeostasis, or in pathological conditions that need to be removed from the body (49-51). Bax and Bcl-2 are strong indicators for apoptosis and are one of the decisive steps in apoptosis (52). A shift in the Bax/Bcl-2 balance in favor of Bax leads to a shift of cytochrome c from the mitochondria towards the stasis and subsequent stimulation of caspase-3 activation (22). Caspase-3, which has a significant role in the apoptotic process, is also known as executioner caspase and has apoptotic properties (53, 54). Researchers (55) reported that Diclo triggered apoptosis by causing caspase-3 activation in different tissues. Varışlı *et al*. (8) reported that Morin exhibited antiapoptotic properties by decreasing Bax and caspase-3 mRNA transcription levels and increasing Bcl-2 mRNA transcription levels in rat testicular tissues. This study determined that Diclo administration in rat testicular tissues showed apoptotic effect by increasing Bax and caspase-3 mRNA transcription levels, which are apoptotic factors, and decreasing Bcl-2 mRNA transcription level, which is antiapoptotic. In the present study, Bax and caspase-3 protein levels were increased in the Diclo group in rat testicular tissues, which may prove that Diclo also affects protein levels. Morin co-administration with Diclo reversed this situation and exhibited antiapoptotic properties at both mRNA transcription and protein levels.

Increased oxidative damage also triggers ER stress injury. ER is a cellular organelle involved in many significant functions (protein synthesis, folding and maturation, and calcium homeostasis) (56). Among the causes of ER stress is the accumulation of unfolded or misfolded proteins in the ER lumen due to an imbalance following physiological or pathological processes (57). ATF-6, PERK, IRE1, GRP78, and CHOP are the most important markers of ER stress damage and increase ER stress (58, 59). In the present study, Diclo caused ER stress by increasing ATF-6, PERK, IRE1, GRP78, and CHOP mRNA transcript levels in rat testicular cells. On the other hand, Morin decreased ER stress damage by decreasing the levels of these parameters.

Increased levels of ROS can cause toxic damage to tissues and promote inflammatory responses (60, 61). The increase of oxygen radicals damages cellular activities by affecting NF-κB pathways (62, 63). NF-κB causes damage to spermatogenesis by increasing both oxidative damage and the release of pro-inflammatory cytokines (8, 64). In this study, NF-κB protein levels increased in response to Diclo toxicity. With morin administration, this situation was reversed; NF-κB protein levels decreased, and inflammatory damage in testicular tissue decreased.

Oxidative stress can also cause damage to testicular tissue Leydig cells and negatively affect the process of spermatogenesis (65). Sertoli cells are the cells involved in the process of spermatogenesis. Leydig cells are responsible for androgen production. When these cells are affected by chemical factors, the hormonal balance can be disrupted, which can have a negative impact on male fertility (66). Decreased testicular cell population dynamics and abnormal testis histology attest to Diclo-induced toxicity. Diclo toxicity may disrupt the microenvironment of Sertoli cells due to degeneration, vacuolation, and apoptosis of spermatogonia, primary spermatocytes, secondary spermatocytes, and spermatids (67). In the present study, thinning of tubule walls, tubular atrophy, and severe edema in the intertubular area were observed in the testicular tissues of the Diclo-treated group. In addition, loss of tubule cells, necrotic and degenerative changes in spermatogonia, and a severe decrease in the number of spermatozoa in the lumens were observed. Morin treatment reduced these damages.

**Table 1:** Primer sequences of rat

Gene	Sequences (5’-3’)	Product length	Accession No
Nrf2	F: TTTGTAGATGACCATGAGTCGC R: TCCTGCCAAACTTGCTCCAT	161	NM_031789.2
HO-1	F: ATGTCCCAGGATTTGTCCGA R: ATGGTACAAGGAGGCCATCA	144	NM_012580.2
NQO1	F: CTGGCCAATTCAGAGTGGCA R: GATCTGGTTGTCGGCTGGAA	304	NM_017000.3
COX-2	F: AGGTTCTTCTGAGGAGAGAG R: CTCCACCGATGACCTGATAT	240	NM_017232.3
ATF-6	F: TCAACTCAGCACGTTCCTGA R: GACCAGTGACAGGCTTCTCT	130	NM_001107196.1
PERK	F: GATGCCGAGAATCATGGGAA R: AGATTCGAGAAGGGACTCCA	198	NM_031599.2
IRE1	F: GCAGTTCCAGTACATTGCCATTG R: CAGGTCTCTGTGAACAATGTTGA	163	NM_001191926.1
GRP78	F: CATGCAGTTGTGACTGTACCAG R: CTCTTATCCAGGCCATATGCAA	143	NM_013083.2
CHOP	F: GAAGCCTGGTATGAGGATCT R: GAACTCTGACTGGAATCTGG	209	NM_001109986.1
Bax	F: TTTCATCCAGGATCGAGCAG R: AATCATCCTCTGCAGCTCCA	154	NM_017059.2
Bcl-2	F: GACTTTGCAGAGATGTCCAG R: TCAGGTACTCAGTCATCCAC	214	NM_016993.2
Caspase-3	F: ACTGGAATGTCAGCTCGCAA R: GCAGTAGTCGCCTCTGAAGA	270	NM_012922.2
-Actin	F: CAGCCTTCCTTCCTGGGTATG R: AGCTCAGTAACAGTCCGCCT	360	NM_031144.3

MDA: Malondialdehyde, GSH: Glutathione, SOD: Superoxide dismutase, GPx: Glutathione peroxidase, CAT: Catalase. Statistical significance (Control vs others: *P<0.05, **P<0.01, and ***P<0.001, Diclo vs others: #*P*<0.05, ##*P*<0.01, and ###*P*<0.001, Diclo+Morin 50 vs Diclo+Morin 100: *P<0.05,**P<0.01, and ***P<0.001) was analyzed using One Way ANOVA

Nrf-2: Nuclear factor erythroid 2-related factor 2, HO-1: Heme oxygenase-1, and NQO1: NAD(P)H Quinone Dehydrogenase 1. Statistical significance (Control vs others: *P<0.05, **P<0.01, and ***P<0.001, Diclo vs others: #*P*<0.05, ##*P*<0.01, and ###*P*<0.001, Diclo+Morin 50 vs Diclo+Morin 100: *P<0.05, **P<0.01, and ***P<0.001) was analyzed using One Way ANOVA.

ATF-6: Activating transcription factor 6, PERK: Protein Kinase RNA-Like ER Kinase, IRE1: Inositol-requiring enzyme 1, GRP-78: Glucose-regulated protein, CHOP: C/EBP homologous protein. Statistical significance (Control vs others: *P<0.05, **P<0.01, and ***P<0.001, Diclo vs others: #*P*<0.05, ##*P*<0.01, and ###*P*<0.001, Diclo+Morin 50 vs Diclo+Morin 100: *P<0.05, **P<0.01, and***P<0.001) was analyzed using One Way ANOVA.

Bax: Bcl-2-associated X, Bcl-2: B-cell lymphoma 2, caspase-3: cysteine-aspartic acid protease-3. Statistical significance (Control vs others: *P<0.05, **P<0.01, and ***P<0.001, Diclo vs others: #*P*<0.05, ##*P*<0.01, and ###*P*<0.001, Diclo+Morin 50 vs Diclo+Morin 100: *P<0.05, **P<0.01, and ***P<0.001) was analyzed using One Way ANOVA.

COX-2: Cyclooxygenase-2. Statistical significance (Control vs others: *P<0.05, **P<0.01, and ***P<0.001, Diclo vs others: #*P*<0.05, ##*P*<0.01, and ###*P*<0.001, Diclo+Morin 50 vs Diclo+Morin 100: *P<0.05, **P<0.01, and ***P<0.001) was analyzed using One Way ANOVA.

NF-κB: Nuclear Factor kappa B, caspase-3: cysteine-aspartic acid protease-3, Bax: Bcl-2-associated X. Statistical significance (Control vs others: *P<0.05, **P<0.01, and ***P<0.001, Diclo vs others: #*P*<0.05, ##*P*<0.01, and ###*P*<0.001, Diclo+Morin 50 vs Diclo+Morin 100: *P<0.05,**P<0.01, and ***P<0.001) was analyzed using One Way ANOVA.

Control group (A,a) and Morin group (B,b): normal histologic appearance of testicular tissue; Diclo group: thinning of seminiferous tubule wall (curved arrow), necrosis and degeneration of spermatogonium (arrowhead), severe edema in interstitial spaces (asterisk) (C,c); Diclo+Morin 50 group: mild edema in interstitial spaces (asterisk) (D, d); Diclo+Morin 100; congestion (arrow) (E, e). H&E, Bar; top row: 100 μm, bottom row: 20 μm

**Table 2:** Effect of diclofenac and Morin on testicular histological parameters

Gene	Sequences (5’-3’)	Product length	Accession No
*Nrf2*	F: TTTGTAGATGACCATGAGTCGC R: TCCTGCCAAACTTGCTCCAT	161	NM_031789.2
*HO-1*	F: ATGTCCCAGGATTTGTCCGA R: ATGGTACAAGGAGGCCATCA	144	NM_012580.2
*NQO1*	F: CTGGCCAATTCAGAGTGGCA R: GATCTGGTTGTCGGCTGGAA	304	NM_017000.3
*COX-2*	F: AGGTTCTTCTGAGGAGAGAG R: CTCCACCGATGACCTGATAT	240	NM_017232.3
*ATF-6*	F: TCAACTCAGCACGTTCCTGA R: GACCAGTGACAGGCTTCTCT	130	NM_001107196.1
*PERK*	F: GATGCCGAGAATCATGGGAA R: AGATTCGAGAAGGGACTCCA	198	NM_031599.2
*IRE1*	F: GCAGTTCCAGTACATTGCCATTG R: CAGGTCTCTGTGAACAATGTTGA	163	NM_001191926.1
*GRP78*	F: CATGCAGTTGTGACTGTACCAG R: CTCTTATCCAGGCCATATGCAA	143	NM_013083.2
*CHOP*	F: GAAGCCTGGTATGAGGATCT R: GAACTCTGACTGGAATCTGG	209	NM_001109986.1
*Bax*	F: TTTCATCCAGGATCGAGCAG R: AATCATCCTCTGCAGCTCCA	154	NM_017059.2
*Bcl-2*	F: GACTTTGCAGAGATGTCCAG R: TCAGGTACTCAGTCATCCAC	214	NM_016993.2
*Caspase-3*	F: ACTGGAATGTCAGCTCGCAA R: GCAGTAGTCGCCTCTGAAGA	270	NM_012922.2
*-Actin*	F: CAGCCTTCCTTCCTGGGTATG R: AGCTCAGTAACAGTCCGCCT	360	NM_031144.3

## Conclusion

Morin showed an ameliorative effect on Diclo-induced testicular toxicity in the present study. In this context, the protective effect of Morin against Diclo-induced testicular toxicity may be mediated by its attenuating effect on oxidative stress injury, inflammatory injury, apoptotic injury, and ER stress injury.

## Declaration

We have not used any AI tools or technologies to prepare this manuscript.
